# Analysis of pre-operative factors affecting range of optimal vaulting after implantation of 12.6-mm V4c implantable collamer lens in myopic eyes

**DOI:** 10.1186/s12886-018-0835-x

**Published:** 2018-07-06

**Authors:** Hun Lee, David Sung Yong Kang, Jin Young Choi, Byoung Jin Ha, Eung Kweon Kim, Kyoung Yul Seo, Tae-im Kim

**Affiliations:** 1 Department of Ophthalmology, International St. Mary’s Hospital, Catholic Kwandong University College of Medicine, Incheon, South Korea; 2 The Institute of Vision Research, Department of Ophthalmology, Yonsei University College of Medicine, Seoul, South Korea; 3 Eyereum Eye Clinic, Seoul, South Korea; 4 Corneal Dystrophy Research Institute, Department of Ophthalmology, Yonsei University College of Medicine, Seoul, South Korea

**Keywords:** 12.6-mm V4c implantable collamer lenses, Postoperative vaulting, Preoperative pupil size, Anterior chamber depth, Axial length

## Abstract

**Background:**

To evaluate clinical factors affecting postoperative vaulting in eyes that had achieved optimal vaulting within the range of 250–750 μm following implantation of 12.6-mm V4c implantable collamer lenses (ICL).

**Methods:**

A total of 236 eyes of 236 patients that had achieved optimal vaulting following implantation of a 12.6-mm V4c ICL were retrospectively analyzed. Associations between postoperative vaulting and age, preoperative anterior chamber depth (ACD), preoperative axial length (AL), preoperative white-to-white diameter, preoperative pupil size, preoperative sulcus-to-sulcus diameter, and preoperative manifest refraction spherical equivalent were investigated using simple regression, stepwise multiple regression, and multinomial logistic regression analyses.

**Results:**

Mean central vaulting at the 6-month follow-up was 519.0 ± 112.8 μm. Variables relevant to postoperative vaulting were, in order of influence, preoperative ACD (β = 0.305, *p* <  0.001), preoperative pupil size (β = 0.218, *p* <  0.001), and preoperative AL (β = 0.171, *p* = 0.006). Low preoperative pupil size was associated with low optimal vaulting (250 to 450 μm), relative to that observed in the mid optimal vaulting group (451 to 550 μm) (odds ratio = 0.532, *P* = 0.021). Increasing preoperative ACD was associated with high optimal vaulting (551 and 750 μm), relative to that observed the mid optimal vaulting group (odds ratio = 6.340, *P* = 0.034).

**Conclusions:**

Myopic eyes with greater preoperative ACD, larger pupil size, and longer AL are predisposed to higher postoperative vaulting following 12.6-mm V4c ICL implantation. Therefore, the extremes of these parameters should be considered when choosing V4c ICL size.

## Background

The implantable collamer lens (ICL), a posterior chamber phakic intraocular lens, effectively correct moderate to high myopia [1, 2]. In addition, the ICL has been reported to be safe and effective for the long-term correction of refractive errors in highly myopic eyes, for which laser vision surgery is not appropriate [3]. Albeit its outstanding benefit, postoperative complications have been reported [4–6]. Most of these complications are associated with vaulting of the lens (i.e., distance between the posterior surface of the ICL and the anterior surface of the crystalline lens). High vaulting conditions lead to increased intraocular pressure and inflammation by causing mechanical contact between the ICL and iris [6, 7]. Pigment dispersion, iris atrophy, secondary glaucoma, and formation of metabolic cataracts have also been associated with high vaulting conditions [8, 9]. Low vaulting conditions have been reported to induce mechanical contact between the ICL and crystalline lens and cause inadequate aqueous circulation in the perilenticular space. Mechanical contact between the ICL (posterior surface) and crystalline lens (anterior surface), as well as impaired circulation of aqueous humor, are considered to play a crucial role in the development of anterior subcapsular cataracts [2, 4, 10].

The recently introduced Visian V4c ICL (STAAR Surgical Company, Monrovia, CA, USA) has been designed with a 360-μm central hole (aquaport) allowing aqueous humor to flow without the need for an iridotomy [11]. The presence of the central hole does not significantly affect the position of the ICL when comparing the V4c ICL and V4b ICL implants [12]. Recent studies have suggested that optimal ICL vaulting ranges from 250 to 750 μm [13–15]. ICL sizing based on sulcus-to-sulcus (STS) diameter, white-to-white (WTW) diameter, and anterior chamber depth (ACD) has been established as the gold standard for achieving optimal vaulting [13, 16, 17]. However, values at the low end of this range (250 μm) may be associated with peripheral crystalline lens contact, while values at the high end of this range (750 μm) may be associated with synechial angle closure. Thus, both optimal vaulting status and the extent of vaulting achieved within the optimal range may be relevant to the selection of ICL size.

Therefore, the present study aimed to analyze factors that influence vaulting in 12.6-mm V4c ICL implanted eyes that had achieved optimal vaulting between 250 and 750 μm. Only one size of V4c ICL was used for the analysis to eliminate any confounding effects of size.

## Methods

Ethical approval for the present retrospective study was obtained from the Institutional Review Board of Yonsei University College of Medicine, Seoul, South Korea (4–2016-0357). The study adhered to the tenets of the Declaration of Helsinki and followed good clinical practice.

Inclusion criteria for the present study were as follows: (i) age 20–45 years; (ii) presence of myopia with a manifest refraction spherical equivalent (MRSE) between − 4.00 and − 20.00 diopters (D); (iii) astigmatism between 0.00 and − 5.00 D; (iiii) eyes that had underwent the implantation of 12.6-mm V4c ICL using standardized techniques performed by one surgeon (DSYK) between August 2013 and February 2016 and achieved optimal vaulting within the range of 250 and 750 μm. Among total 293 eyes, 7 eyes (3%) showed vaulting smaller than 250 μm and 32 eyes (11%) showed vaulting larger than 750 μm. Patients were excluded from the analysis if they had keratoconus, previous ocular or intraocular surgery, acute or chronic corneal infection (bacterial and fungal), corneal inflammation (keratitis, herpes zoster, ocular herpes, and Stevens-Johnson syndrome), glaucoma, cataract, uveitis, retinal detachment, macular degeneration (age-related and myopic), an endothelial cell density < 2000 cells/mm^2^, or an ACD from the endothelium < 2.8 mm. One eye from each patient was included in the analysis using randomization table.

All patients underwent complete ophthalmic examinations, including uncorrected and corrected distance visual acuity (Snellen lines), manifest refraction with the cross-cylinder technique following retinoscopy, slit-lamp microscopy (Haag-Streit, Gartenstadtstrasse, Köniz, Switzerland), tonometry (noncontact tonometer; NT-530, Nidek Co., Ltd., Aichi, Japan), autokeratometry (ARK-530A; Nidek Co., Ltd.), automated pupillometry (VIP-200; Neuroptics Inc., Irvine, CA, USA), noncontact specular microscopy (SP-3000P, Topcon Corporation, Tokyo, Japan), ultrasound pachymetry for measurement of central corneal thickness via contact method, A-scan ultrasonography, Visante optical coherence tomography (OCT; Carl Zeiss Meditec AG, Jena, Germany) for measurement of ACD (vertical distance from the central corneal endothelium to the anterior lens capsule) and horizontal WTW, and fundus examination. Ultrasound biomicroscopy (UBM) was performed to measure the horizontal STS diameter after instillation of proparacaine (Alcaine; Alcon, Fort Worth, TX, USA) under standard room lighting conditions. An independent physician performed all ultrasound biomicroscopy examinations using the UBM equipped with a 50 MHz transducer.

Six months after implantation, the central vaulting of the ICL over the crystalline lens was measured in the non-accommodative state using the Visante OCT. Central vaulting was defined as the distance between the posterior surface of the ICL and the anterior surface of the crystalline lens at the center of the implant. Each measurement was performed three times by one physician, and the average of the three measurements was used in the analysis. A non-accommodative state was ensured by asking patients to avoid visual display terminal equipment or books for at least 3 h before examinations [18]. Measurements were taken while the patient fixated on a collimated light-emitting diode (focus at infinity) in a room with a luminance of 2 lux [19].

### Surgical procedure

The surgery was performed through a 3.0-mm clear superior corneal incision after dilation of the pupil with 0.5% phenylephrine and 0.5% tropicamide (Mydrin-P, Santen Pharmaceutical Co. Ltd., Osaka, Japan) under topical anesthesia. The anterior chamber was filled with 1% sodium hyaluronate (Healon; Abbott Medical Optics, Santa Ana, CA, USA), which was removed completely by manual irrigation and aspiration at the end of surgery. Emmetropia was the target refraction in all cases. The ICL was then inserted using an injector cartridge and correctly positioned. The V4c ICL power calculations were performed according to the manufacturer’s guidelines using a modified vertex formula [20]. Following surgery, a topical antibiotic (Vigamox; Alcon Laboratories) and loteprednol etabonate 0.5% (Lotemax; Bausch & Lomb, Rochester, NY, USA) were applied four times a day for one week. After the first week, loteprednol etabonate 0.5% was replaced with 0.1% fluorometholone (Flumetholon; Santen Pharmaceutical). All eye drops were continued four times a day for one month.

### Statistical analysis

The Kolmogorov-Smirnov test was used to confirm the normality of the data. Simple regression analyses and stepwise multiple regression analyses were performed to investigate the association between the amount of vaulting at 6 months after surgery and several preoperative variables. The dependent variable was the central vaulting of the ICL over the crystalline lens using the Visante OCT. Independent variables included patient age, gender, preoperative MRSE, pupil size, WTW, STS, ACD, and axial length (AL). Analysis of variance (ANOVA) followed by Bonferroni post hoc testing were performed to examine differences among subgroups, which were based on the extent of postoperative vaulting (250 to 450 μm: low optimal vaulting; 451 to 550 μm: mid optimal vaulting; and 551 and 750 μm: high optimal vaulting). Multinomial logistic regression analysis was performed to ascertain the effects of age, gender, preoperative manifest refraction spherical equivalent (MRSE), pupil size, WTW, STS, ACD, and AL on vaulting outcomes. Statistical analyses were performed using SPSS version 22.0 software (IBM Corp., Armonk, NY, USA). A *p*-value less than 0.05 was considered statistically significant.

## Results

The mean patient age was 28.2 ± 5.1 (range 20–44) years, and 71% (168/236) of the patients were females. Table 1 summarizes the baseline clinical characteristics of the 236 patients and descriptive data for preoperative variables. The mean ICL power was − 11.2 ± 2.2 (range − 5.5 to − 18.0) D. The mean central vaulting of the ICL at 6 months after surgery was 519.0 ± 112.8 μm (range 250–740). All surgeries were uneventful, and no intraoperative complications were noted. No contact between the ICL and crystalline lens was observed at either the center or periphery of the implant in any patient during the follow-up period.

**Table 1:** Baseline clinical characteristics of the study eyes (236 eyes)

Characteristics	Mean ± SD	Range
Age (yrs)	28.2 ± 5.1	20 to 44
Gender (male/female) (%)	29/71	
Laterality (right/left) (%)	48/52	
Preoperative CDVA (Snellen lines)	0.97 ± 0.09	1.00 to 1.20
Preoperative sphere (D)	−8.48 ± 2.28	−3.50 to −16.75
Refractive cylinder (D)	−1.41 ± 0.84	−4.75 to 0.00
Preoperative MRSE (D)	−9.19 ± 2.36	−4.00 to − 19.13
Preoperative pupil size (mm)	7.16 ± 0.64	4.70 to 8.60
Preoperative WTW (mm)	11.46 ± 0.28	10.85 to 12.80
Preoperative STS (mm)	11.65 ± 0.26	10.81 to 12.22
Preoperative ACD (mm)	3.35 ± 0.20	2.88 to 3.89
Preoperative AL (mm)	27.18 ± 1.16	23.88 to 30.82
Preoperative ECD (cells/mm^2^)	3018.4 ± 301.0	2229 to 3747

*CDVA* corrected distance visual acuity, *MRSE* manifest refraction spherical equivalent, *D* diopters, *WTW* white-to-white, *STS* sulcus-to-sulcus, *ACD* anterior chamber depth, *AL* axial length, *ECD* endothelial cell density, *SD* standard deviation

Table 2 shows the simple regression analysis results between postoperative vaulting and eight variables. There was significant association between postoperative vaulting and preoperative pupil size (*P* = 0.004), and between postoperative vaulting and preoperative ACD (*P* <  0.001). According to Table 3 showing the results of the stepwise multivariate regression analysis, the explanatory variables relevant to vaulting were preoperative ACD (*P* <  0.001, standardized partial regression coefficient [β] = 0.305), preoperative pupil size (*P* <  0.001, β = 0.218), and preoperative AL (*P* = 0.006, β = 0.171). The multiple regression equation was expressed as follows: central vaulting (μm) = − 0.784 + (0.171 × preoperative ACD) + (0.038 × preoperative pupil size) + (0.017 × preoperative AL). The standardized partial regression coefficient was calculated to determine the magnitude of the influence of each variable. Thus, preoperative ACD was the most relevant variable, followed by preoperative pupil size and AL (Table 3). Higher central vaulting was observed in eyes with greater preoperative ACD, larger pupil size, or longer AL.

**Table 2:** Simple regression analysis result between the postoperative vaulting and eight variables

	*R* ^*2*^	*P*
Age (yrs)	0.012	0.092
Gender	0.003	0.370
MRSE (D)	0.011	0.103
Preoperative pupil size (mm)	0.036	0.004
Preoperative WTW (mm)	0.001	0.813
Preoperative STS (mm)	0.008	0.169
Preoperative ACD (mm)	0.072	< 0.001
Preoperative AL (mm)	0.015	0.057

*MRSE* manifest refraction spherical equivalent, *D* diopters, *WTW* white-to-white, *STS* sulcus-to-sulcus, *ACD* anterior chamber depth, *AL* axial length

**Table 3:** Results of stepwise multiple regression analysis to select variables relevant to central vaulting in 12.6-mm V4c implantable collamer lens implanted eyes with postoperative optimal range of vaulting

Variables	Partial regression coefficient (B)	Standardized partial regression coefficient (*β)*	*P*	*R* ^*2*^
			< 0.001	0.144
Preoperative ACD (mm)	0.171	0.305	< 0.001	
Preoperative pupil size (mm)	0.038	0.218	< 0.001	
Preoperative AL (mm)	0.017	0.171	0.006	
Constant	−0.784			

Variables in the table are ordered according to the strength of the contribution, which was based on the standardized partial regression coefficient (*β)*

*ACD* anterior chamber depth, *AL* axial length

**Fig. 1:**
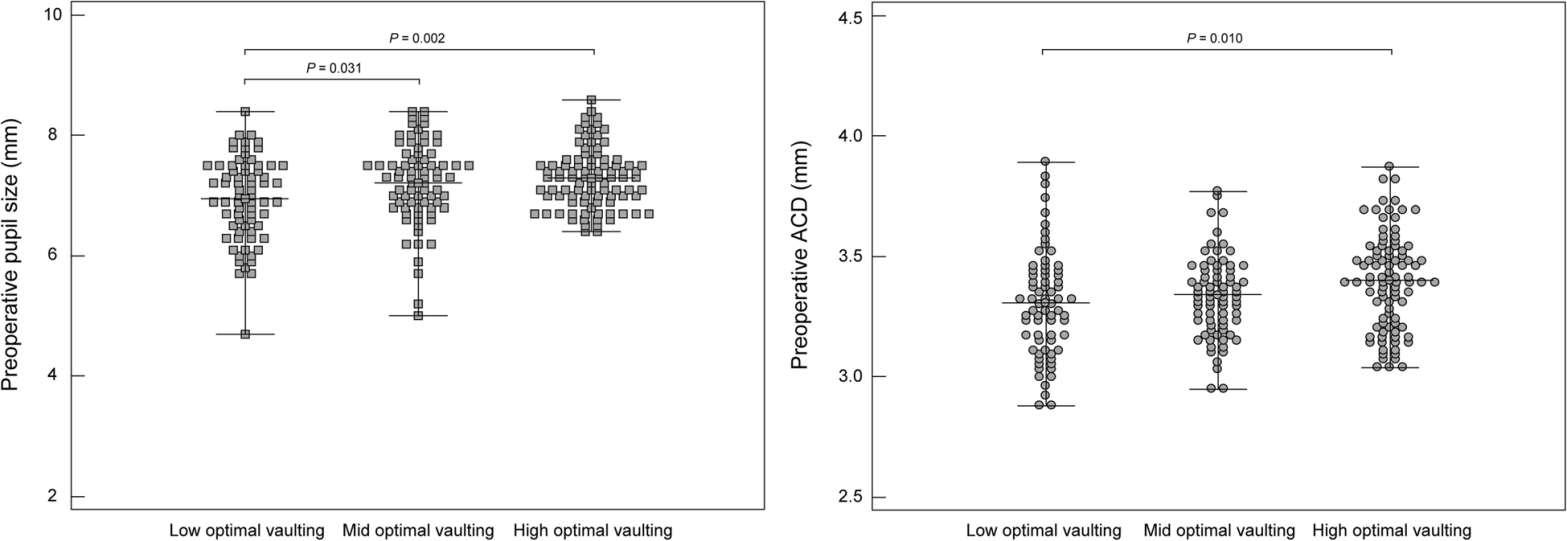
Subgroup analyses according to degree of postoperative vaulting. Low optimal vaulting, 250 to 450 μm; mid optimal vaulting, 451 to 550; high optimal vaulting, 551 and 750 μm. ACD = anterior chamber depth. Error bars represent range

We also performed subgroup analyses according to degree of postoperative vaulting. We observed a significant difference in pupil size between the low optimal vaulting and mid optimal vaulting groups (*P* = 0.031), and between the low optimal vaulting and high optimal vaulting groups (*P* = 0.002, Fig. 1). Significant differences in ACD were also observed between the low optimal vaulting and high optimal vaulting groups (*P* = 0.010) (Table 4 and Fig. 1). Similar to findings observed for ACD, our results suggested that smaller pupil diameters were associated with low optimal vaulting values. Multinomial logistic regression analysis revealed that low preoperative pupil size was associated with low optimal vaulting (odds ratio = 0.532, *P* = 0.021, low vaulting vs mid vaulting), and that increasing preoperative ACD was associated with high optimal vaulting (odds ratio = 6.340, *P* = 0.034, mid vaulting vs high vaulting).

**Table 4:** Subgroup analysis of preoperative clinical factors in 12.6-mm V4c implantable collamer lens implanted eyes with postoperative optimal range of vaulting according to degree of postoperative vaulting

Characteristics	250 to 450 μm (*n* = 70)	451 to 550 μm (*n* = 75)	551 and 750 μm (*n* = 91)	*P*
Age (yrs)	29.3 ± 5.7	27.8 ± 5.4	27.6 ± 4.3	0.076
Preoperative MRSE (D)	−8.79 ± 1.90	−9.28 ± 2.24	− 9.41 ± 2.74	0.237
Preoperative pupil size (mm)	6.95 ± 0.70^a,b^	7.22 ± 0.67^a^	7.29 ± 0.50^b^	0.002
Preoperative WTW (mm)	11.47 ± 0.29	11.46 ± 0.29	11.44 ± 0.26	0.874
Preoperative STS (mm)	11.64 ± 0.30	11.61 ± 0.25	11.68 ± 0.24	0.251
Preoperative ACD (mm)	3.31 ± 0.22^c^	3.34 ± 0.17	3.40 ± 0.20^c^	0.010
Preoperative AL (mm)	26.98 ± 1.02	27.19 ± 1.15	27.32 ± 1.25	0.184

*MRSE* manifest refraction spherical equivalent, *D* diopters, *WTW* white-to-white, *STS* sulcus-to-sulcus, *ACD* anterior chamber depth, *AL* axial length

^a^*P* = 0.031(two-way ANOVA, Bonferroni post hoc test)

^b^*P* = 0.002 (two-way ANOVA, Bonferroni post hoc test)

^c^*P* = 0.010 (two-way ANOVA, Bonferroni post hoc test)

## Discussion

In the present study, we demonstrated that preoperative ACD, followed by preoperative pupil size and preoperative AL, significantly influenced postoperative vaulting following 12.6-mm V4c ICL implantation in eyes that had achieved optimal vaulting within the range of 250–750 μm. Our findings are consistent with those of a recent study in which stepwise multiple regression analysis of patient age, preoperative refraction, WTW, horizontal and vertical STS, ACD, AL, keratometric readings, and ICL power revealed that ACD was the only factor significantly associated with vaulting in eyes that had undergone implantation of V4c ICL [21]. This previous study included 39 eyes of 39 patients that had undergone implantation with wide range of V4c ICL overall diameters (12.1-mm, 12.6-mm, 13.2-mm, or 13.7-mm) [21]. In recent study identifying factors associated with the unexpected vaulting in eyes that had undergone implantation of V4c ICL, authors concluded that smaller sized ICL should be considered in patients with shallow ACD [22]. Postoperative vaulting was also associated with preoperative ACD, WTW, and horizontal and vertical STS in eyes that had undergone implantation of a V4 or V4c ICL [16]. In that study, the presence of a central hole and the size of the implanted V4 or V4c ICL did not significantly influence postoperative vaulting. Furthermore, in a previous study that aimed to identify ocular and lens parameters predictive of vaulting after ICL implantation, eyes with a shallower ACD and/or a smaller WTW exhibited significantly lower postoperative vaulting [23]. In our study, because ICL size was limited to only 12.6-mm, the WTW and STS were relatively less important factors predictive of vaulting when compared with the ACD.

Several previous studies have reported that changes in pupil size in response to certain stimuli, such as photopic light exposure or during accommodation, are associated with postoperative vaulting [24–26]. In a previous study, we demonstrated that pupil constriction in response to photopic light exposure creates an antero-posterior vector via iris constriction, in which the iris exerts pressure on the ICL [23]. Because the V4C ICL has a central hole, pressure equilibrium is quickly achieved between the anterior and posterior surfaces of the ICL, facilitating this process (fountain effect of aquaport). In other words, the net effect of pushing the ICL closer to the crystalline lens is produced, subsequently decreasing central vaulting. Furthermore, in a recent study of early postoperative changes in vaulting and pupil size in eyes that had undergone V4c ICL implantation, the authors reported a significant association between the changes in vaulting and those in pupil size from 1 day to 1 month postoperatively, concluding that pupil movement significantly influences postoperative vaulting [21]. These results indicate that postoperative vaulting is influenced by status of the anterior chamber. In the present study, we demonstrated that, once ICL size has been determined based on STS and WTW measurements, preoperative pupil size significantly influences postoperative vaulting. We speculate that, in association with changes in aqueous humor dynamics via the central hole in the V4c ICL, preoperative pupil size may influence circumstances at the anterior region of the eye, especially the region between the posterior cornea and anterior crystalline lens surface. Smaller pupils may exert greater pressure on the ICL than larger pupils, consequently decreasing vaulting. Indeed, our findings demonstrated that smaller preoperative pupil size was associated with low optimal vaulting, relative to values observed in the mid optimal vaulting group (odds ratio = 0.532, *P* = 0.021). Therefore, once ICL size has been determined based on preoperative ACD, AL, WTW, and STS measurements, surgeons should also consider preoperative pupil size when predicting vaulting outcomes.

Preoperative AL was significantly associated with postoperative vaulting in Stepwise multiple regression analysis. To the best of our knowledge, no previous reports have suggested that preoperative AL significantly influences postoperative vaulting. Considering that ACD is positively correlated with AL in both eyes with normal and long AL, preoperative AL may be relevant to postoperative vaulting [27].

The present study has several limitations, including its retrospective design and a relatively short follow-up duration of 6 months. However, to our knowledge, our study is the first to investigate the relationship between postoperative vaulting and preoperative pupil size in 12.6-mm V4c ICL implanted eyes with optimal range of vaulting between 250 and 750 μm. Further study investigating the effect of preoperative pupil size on aqueous humor dynamics in V4c ICL implanted eyes is warrant.

## Conclusions

Our findings demonstrate that, among the studied variables, preoperative ACD most significantly influenced postoperative vaulting in 12.6-mm V4c ICL implanted eyes with optimal range of vaulting, followed by preoperative pupil size and AL. Therefore, surgeons should take into account preoperative ACD, pupil size, and AL when considering implantation of a 12.6-mm V4c ICL, particularly in patients exhibiting extreme values, for whom size adjustments may be required.

## Abbreviations

ACD: Anterior chamber depth
AL: Axial length
ICL: Implantable collamer lenses
MRSE: Manifest refraction spherical equivalent
STS: Sulcus-to-sulcus
WTW: White-to-white
